# Association between homocysteine and coronary artery disease—trend over time and across the regions: a systematic review and meta-analysis

**DOI:** 10.1186/s43044-024-00460-y

**Published:** 2024-02-27

**Authors:** Sumit V. Unadkat, Bijaya K. Padhi, Aparna Varma Bhongir, Aravind P. Gandhi, Muhammad Aaqib Shamim, Neelam Dahiya, Prakasini Satapathy, Sarvesh Rustagi, Mahalaqua Nazli Khatib, Abhay Gaidhane, Quazi Syed Zahiruddin, Ranjit Sah, Hashem Abu Serhan

**Affiliations:** 1 Department of Community Medicine, M. P. Shah Government Medical College, Jamnagar, Gujarat India; 2 Department of Community Medicine and School of Public Health, Postgraduate Institute of Medical Education and Research, Chandigarh, 160012 India; 3 Department of Biochemistry, All India Institute of Medical Sciences, Bibinagar, Hyderabad India; 4 Department of Community Medicine, All India Institute of Medical Sciences, Nagpur, 441108 India; 5 Department of Pharmacology, All India Institute of Medical Sciences, Jodhpur, 342005 India; 6 Global Center for Evidence Synthesis, Chandigarh, 160036 India; 7 Department of Cardiology, Postgraduate Institute of Medical Education and Research, Chandigarh, 160012 India; 8 Center for Global Health Research, Saveetha Medical College and Hospital, Saveetha Institute of Medical and Technical Sciences, Saveetha University, Chennai, India; 9 School of Pharmacy, Graphic Era Hill University, Dehradun, 248001 India; 10 School of Applied and Life Sciences, Uttaranchal University, Dehradun, Uttarakhand India; 11 Division of Evidence Synthesis, Global Consortium of Public Health and Research, DMIHER, Wardha, India; 12 Jawaharlal Nehru Medical College, One Health Centre (COHERD), Datta Meghe Institute of Higher Education, Wardha, India; 13 Division of Evidence Synthesis, School of Epidemiology and Public Health and Research, Jawaharlal Nehru Medical College, Datta Meghe Institute of Higher Education, Wardha, India; 14 Tribhuvan University Teaching Hospital, Kathmandu, 46000 Nepal; 15 Department of Clinical Microbiology, DY Patil Medical College, Hospital and Research Centre, DY Patil Vidyapeeth, Pune, 411000 Maharashtra India; 16 Department of Ophthalmology, Hamad Medical Corporation, Doha, Qatar

**Keywords:** Coronary artery disease, Acute coronary syndrome, Homocysteine, Systematic review, Meta-analysis

## Abstract

**Background:**

The association of homocysteine with coronary artery disease (CAD) has been explored previously with mixed findings. The present Systematic Review and Meta-Analysis (SRMA) has assessed the pooled estimate of association between homocysteine (Hcy) and CAD, and its variation over the period and geography.

**Methods:**

Systematic literature search was done in PubMed, Scopus and Cochrane to identify the observational studies that have reported mean Hcy among cases (CAD) and control. The SRMA was registered in PROSPERO (ID-CRD42023387675)**.**

**Results:**

Pooled standardized mean difference (SMD) of Hcy levels between the cases and controls was 0.73 (95% CI 0.55–0.91) from 59 studies. Heterogeneity was high (I^2^ 94%). The highest SMD was found among the Asian studies (0.85 [95% CI 0.60–1.10]), while the European studies reported the lowest SMD between the cases and controls (0.32 [95% CI 0.18–0.46]). Meta-regression revealed that the strength of association was increasing over the years (Beta = 0.0227, *p* = 0.048).

**Conclusions:**

Higher homocysteine levels might have a significant association with coronary artery diseases, but the certainty of evidence was rated low, owing to the observational nature of the studies, high heterogeneity, and publication bias. Within the population groups, Asian and African populations showed a greater strength of association than their European and American counterparts, and it also increased over the years.

**Supplementary Information:**

The online version contains supplementary material available at 10.1186/s43044-024-00460-y.

## Background

Cardiovascular disease (CVD) is a major public health and clinical problem across the world. Though mortality due to CVD is decreasing in developed countries, the proportion of deaths due to coronary heart disease (CHD) is increasing (of all the deaths among people over 35 years of age, around one-third are due to CHD) [1]. Over ninety percent of CAD events occur in individuals with at least one risk factor. Among the risk factors, few are non-modifiable, like increasing age, male gender, history of premature CAD among first-degree family members, and some genetic factors, while many are modifiable or preventable risk factors like hypertension, high fasting plasma blood glucose, obesity or overweight, physical inactivity, dyslipidemia (high low-density lipoprotein and low high-density lipoprotein) cigarette smoking, stress, suboptimal diet, etc. [2] But in recent years, studies have reported that known classical risk factors can explain only half to one-third of atherosclerotic vascular events [3]. Certain novel potential risk factors that are being studied and hypothesized are high-sensitivity C-reactive protein, lipoprotein (a), plasma fibrinogen, plasma homocysteine, plasminogen inhibitor type I, endogenous tissue plasminogen activator (tPA), estrogen deficiency, microalbuminuria, increased levels of the leukocyte enzyme myeloperoxidase, etc. [2, 3]

Homocysteine (Hcy) has been studied as an independent risk factor for vascular disease. Researchers across the world are interested in finding out its association with CAD, cerebrovascular disease, and peripheral vascular disease. Fasting plasma Hcy concentrations between 5 and 15 µmol/L are considered normal [3]. The correlation between hyperhomocysteinemia and atherosclerotic disease was first detected more than 50 years ago by McCully in 1969 [4]. One of the earlier meta-analysis done by Boushey et al. concluded that a 5 µmol/L increment in total homocysteine level and 0.5 µmol/L (20 mg/dL) increase in serum cholesterol elevate the similar risk of CAD [5]. Arnesen et al. observed a positive relationship between homocysteine and the risk of myocardial infarction in their large prospective study [6]. Schnyder G. et al. suggested that total homocysteine, along with age and gender, strongly predicts the severity of coronary artery disease and should be assessed for the CVD risk profile of patients as an independent cardiovascular marker [7]. On the contrary, a few studies did not find a significant association between homocysteine and coronary artery disease [8–10]. Thus, the studies have reported mixed findings in terms of the relationship between plasma homocysteine and cardiovascular disease. Preliminary systematic search showed that previously published SRMAs in 2008 [11] and 2022 [12] reported a significant association between Hcy and CHD. However, these analyses had the following limitations: The SRMA published in 2008 included studies representing population from North America and European regions only, undertook Medline and Cochrane database search only [11], while three primary database search is recommended for SRMAs [13]. The outcomes of the included studies were also diverse like any CHD event including CHD death, MI, revascularization procedures, CVD death or stroke [11]. In the 2022 SRMA, the search strategies (database specific), inclusion–exclusion criteria and the outcomes were not comprehensively defined and/or published. Publication bias was not assessed quantitatively, and outlier determination was not done [12]. Certainty of the evidence was not determined [11, 12]. Since studies considered different cut-off values of Hcy based on median, tertiles, quartiles or quintiles, risk ratio (RR) was estimated for each study based on assumed log-linear association between CHD risk and Hcy. So, actual RR of the studies was not used in meta-analysis. Based on the above critical gaps identified, we undertook the index meta-analysis to determine the association between Hcy and the CAD by adopting the following comprehensive and transparent methodology.

## Methods

### Systematic search

The index SRMA adhered to the PRISMA guidelines (Additional file 1: Table S1). The research question for the index SRMA was: What is the association between the plasma homocysteine level and the occurrence of coronary artery disease? which is elaborated in Additional file 1: Table S2. An extensive literature search was done in various databases: PubMed, Scopus and Cochrane. The authors also searched Google Scholar to find other related articles. The base search strategy was formulated for PubMed. The same search strategy was used for the rest of the database as per the required format (Additional file 1: Table S3). We also reviewed the references of the eligible articles and found more studies that could be included in this systematic review and meta-analysis.

### Selection of study

After the removal of duplicates, screening of the studies was done based on the criteria elaborated in Additional file 1: Table S2. Titles and abstracts were screened for the outcome of interest, i.e., Acute Coronary Syndrome or Coronary Artery Disease, and the exposure of interest, i.e., total plasma homocysteine concentration. Through title and abstract screening, observational studies like cross-sectional, case–control and nested case–control studies were identified where the case group represents acute coronary syndrome (ACS) or CAD patients and the control group comprises healthy individuals or patients free from coronary artery disease and exposure, i.e., plasma homocysteine level, has been measured. This initial title-abstract screening of the studies was done independently by two reviewers (SVU and AG). Full-text articles of these short-listed studies were retrieved and again screened by the same two authors (SVU and AG) against predefined study eligibility criteria. At any level, discrepancies about the eligibility of the study were discussed and resolved by the reviewers, and adjudication was sought from the third reviewer (BKP), if consensus was not achieved between the two reviewers.

### Data extraction

All the relevant data were extracted independently by the two authors (SVU and AG). For that, a common data extraction format was prepared using Microsoft Excel. Information like the name of the author, publication year, study type, number of cases and controls, and characteristics of cases and controls were retrieved. Information about exposure, i.e., plasma homocysteine, was collected, like mean and SD among case and control groups, fasting or non-fasting blood samples and methods of homocysteine estimation. If the data were reported as a mean and standard error in the study, then SD was calculated. Other relevant information was also recorded, like the mean age, age group and gender of the study participants.

### Assessment of the quality of the studies

To assess the study quality, the National Heart, Lung, and Blood Institute (NHLBI) tool was used, which was developed for observational studies [14]. Two reviewers assessed the quality of the studies and third reviewer resolved if any discrepancy or disagreement raised. Studies fulfilling at least 75% criteria were labeled good-quality studies, while those with 50% to 75% were fair, and less than 50% were considered poor-quality studies.

### Data analysis

The pooled estimates of the outcomes, along with a 95% confidence interval (95% CI), were measured in terms of standardized mean difference (SMD). I^2^ statistics were applied to assess the heterogeneity of studies, and I^2^ > 50% was considered substantial to high heterogeneity [15]. If I^2^ > 50% was found, a random-effects model (the Der Simonian and Laird method) [16] was applied. The prediction interval of the pooled estimate was determined based on the Tau^2^ statistics [17]. Heterogeneity was explored by undertaking subgroup analyses based on the following variables: case group (ACS/CAD), geography (continent of study), gender (males and females/males only), type of blood sample (fasting or non-fasting), and study period. Assessment of publication bias was planned by means of the Funnel plot, Doi plot, and LFK index if more than ten studies were found eligible for meta-analysis. Baujat plot and influential plots were done to identify the influential studies. A leave-one-out analysis was planned to estimate the impact of each study on the pooled outcome estimate and the heterogeneity. A sensitivity analysis was planned after eliminating the poor-quality studies and influential studies. A p-value of < 0.05 was deemed significant.

The meta-analysis was performed using R statistical software (version 4.2.2 (2022–10–31 urct) following the standard codes [18]. Other R packages used were ‘meta’ (version 6.2–0) and ‘metasens’ (version 1.5–2).

### Certainty in the evidence

The study evaluated and summarized the pooled estimate's certainty for each outcome using the Grading of Recommendations, Assessment, Development, and Evaluations (GRADE) methodology [19].

The systematic review and meta-analysis was registered with PROSPERO (ID-CRD42023387675).

## Results

### Eligible studies

**Fig. 1:**
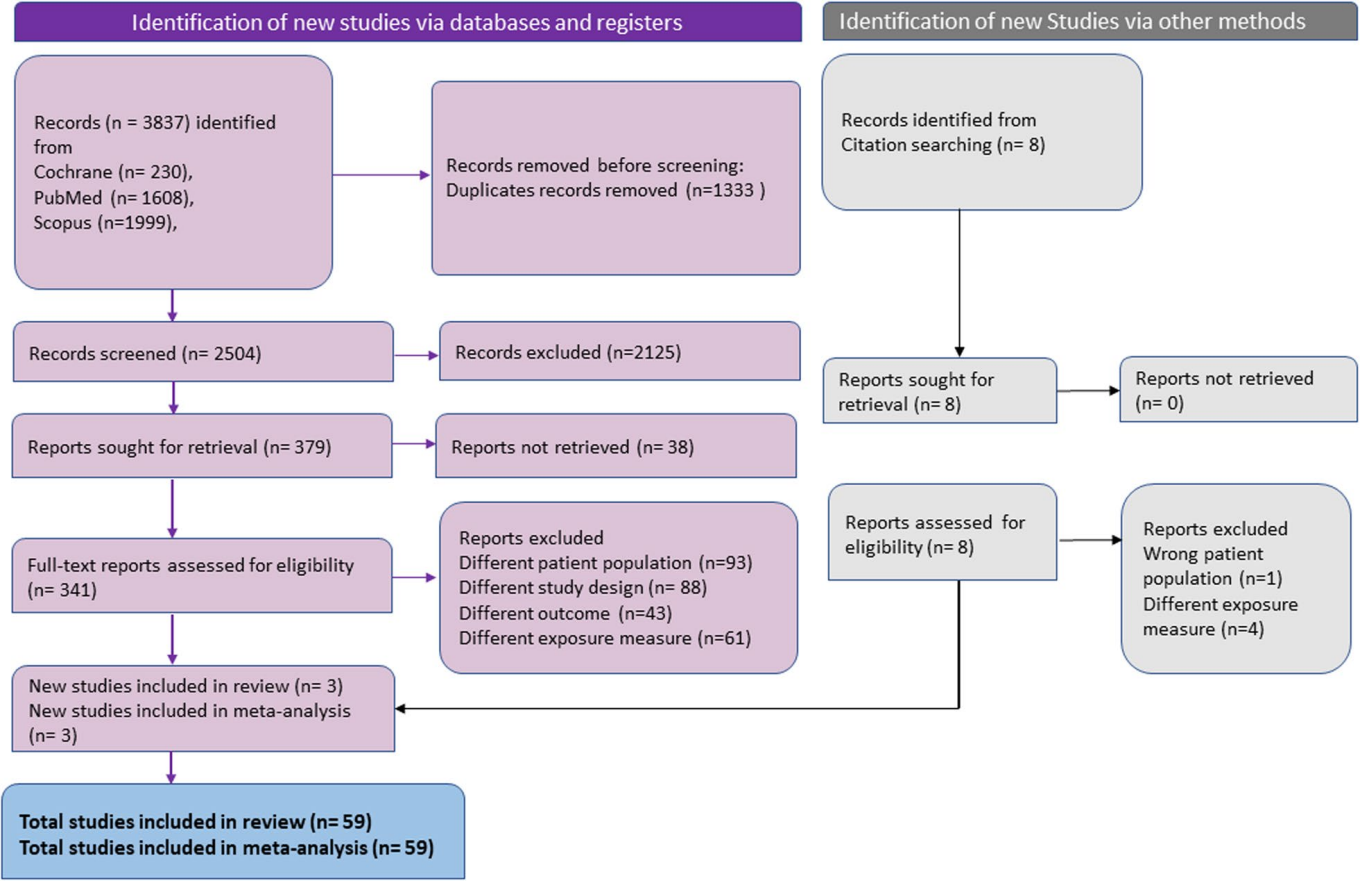
PRISMA flowchart of the study

The databases were thoroughly searched, resulting in a collection of 2504 distinct records. These records were then subjected to title-abstract screening, leading to the identification of 379 studies that were deemed suitable for a comprehensive review. After conducting a thorough examination of the full texts of these studies, 59 of them were determined to be eligible for data extraction and were thus included in the systematic review and meta-analysis (Fig. 1).

### Characteristics of the included studies

The included studies were conducted from 1990 to 2022. The sample size of the studies ranged from 28 to 875 patients in the CAD group and 15 to 2914 controls. Case–control study design was reported in 46 of the 59 studies, while 12 of the 59 were cross-sectional studies and one was a nested case–control study. Ten of the studies included only male participants. Most of the studies were conducted among individuals of any age group (41/59). The majority of the studies were conducted in India-12 [20–31], followed by Turkey-8 [32–39], Tunisia-7 [40–46], Pakistan-5 [47–51], United States of America (USA)-4 [52–55], Taiwan-4 [56–59], China-3 [60–62], Iran-2 [63, 64], and United Kingdom (UK)-2 [65]. Palestine [66], Norway [67], Indonesia [68], South Korea [69], Switzerland [70], Poland [71], France [72], Japan [73], Canada [74], Germany [75], Cyprus [76], and Greece [77] that reported one study each. According to the diagnosis of the cases, 30/59 studies included ACS patients, 16/59 studies included CAD patients with ≥ 50% occlusion, and 13/59 studies included CAD patients without mentioning the exact diagnostic criteria. Homocysteine levels were measured in the fasting blood samples in 49 studies and in non-fasting samples in three studies. Seven studies did not specify the fasting status of the sample (Table 1).

**Table 1:** Characteristics of the Individual studies included in the meta-analysis (n = 59)

Author (year)	Country	Study design	Case group	No. of participants		Age (mean [SD]) years	Gender (% of male participants)	Homocysteine mean (SD)		p value
				Cases	Controls			Cases	Controls	
Abraham R et al. [20]	India	Case–control	ACS	70	70	Cases-56.67 (11.71) Controls-53.01 (12.46)	Cases-Male-84% Controls-Male-75.7%	22.81 (13.9)	7.77 (7.3)	p < 0.001
Akyurek et al. [32]	Turkey	Case–control	ACS	76	56	Cases-38.7 (6.9) Controls-34.6 (10.2)	Cases-Male-73.6% Controls-Male-67.8%	19 (3.6)	15.8 (4.2)	*p* = 0.008
Alawneh I et al. [66]	Palestine	Case–control	CAD ≥ 50%	84	81	Cases-57.63 (11.1) Controls-46.23 (14.65)	Cases-Male-77.4% Controls-Male-61.7%	11.91 (7.63)	9.79 (5.74)	*p* = 0.04
Angeline T et al. [21]	India	Case–control	ACS	100	100	Cases and controls were < 48 years	Cases-Not mentioned Controls-Male-100%	32.35 (10.3)	13.62 (3.56)	p < 0.001
Aydin M et al. [33]	Turkey	Case–control	CAD ≥ 50%	235	268	Cases-60.68 (11.07) Controls 59.24 (10.79)	Cases-Male-73.19% Controls-Male-73.51%	15.89 (9.89)	9.82 (2.76)	p < 0.001
Azhar I et al. [47]	Pakistan	Case–control	ACS	30	30	Cases and control were between 20–45 years	Proportion not mentioned	18.1 (5.3)	14.7 (4.93)	*p* = 0.013
Bahri R et al. [40]	Tunisia	Case–control	CAD	50	50	Not mentioned	Not mentioned	16.6 (4.58)	12.93 (1.12)	p < 0.001
Bahulikar A et al. [24]	India	Case–control	ACS	145	145	Cases-58.25 (12.28) Controls-57.27 (11.42)	Cases-Male-58.62% Controls-Male-60.69%	14.69 (6.68)	12 (3.63)	p < 0.01
Bhagwat VR et al. [25]	India	Cross-sectional	ACS	42	50	Cases and controls were between 20–50 years	Cases-Male-68% Controls-Male-60%	32.48 (18.99)	10.76 (2.77)	p < 0.001
Bozkurt A et al. [34]	Turkey	Cross-sectional	CAD ≥ 50%	195	146	Cases-52.3 (9.8) Controls-50.2 (9.6)	Cases-Male-73.33% Controls-Male-64.38%	16.4 (7.4)	13.2 (3.6)	p < 0.001
Bozkurt E et al. [35]	Turkey	Case–control	CAD	156	35	Cases-56 (11) Controls-55 (10)	Cases-Male-69% Controls-Male-66%	15.59 (5.7)	9.24 (1.5)	p < 0.001
Chalghoum A et al. [41]	Tunisia	Case–control	ACS	157	142	Cases-64.8 (11.7) Controls-56.8 (9.4)	Cases-Male-77% Controls-Male-78.2%	24.4 (12.5)	7.4 (2.5)	p < 0.0001
Chambers JC et al. [65]	UK	Case–control	CAD	230	424	Cases-56 (6) Controls-50 (7)	Only male participants	11.2 (4.6)	10.2 (3.3)	p < 0.01
Chambers JC et al. [65]	UK	Case–control	CAD	224	381	Cases-52 (7) Controls49 (6)	Only male participants	12 (5)	10.8 (3.8)	p < 0.01
Chen CJ et al. [56]	Taiwan	Cross-sectional	ACS	56	17	Cases-58.1 (13) Controls-57.5 (11)	Cases-Male-77% Controls-Male-65%	8.4 (2.2)	7.6 (1.9)	*p* = 0.142
Cheng ML et al. [57]	Taiwan	Case–control	CAD	86	89	Cases-62.9 (10.1) Controls-60.12 (9.13)	Cases-Male-74.4% Controls-Male-47.2%	9.94 (3.71)	8.27 (1.74)	p < 0.05
Christensen B et al. [67]	Norway	Case–control	ACS	107	103	Cases-62.1 Controls-62.7	Cases-Male-70.9% Controls-Male-71%	12 (4.2)	10.9 (3)	p < 0.05
Chua S et al. [58]	Taiwan	Case–control	ACS	178	30	Cases-60.2 (11.9) Controls-54 (9)	Cases-Male-82.6% Controls-Male-66.7%	10.5 (3.3)	8.3 (2.4)	*p* = 0.0004
Dalery K et al. [74]	Canada	Case–control	CAD ≥ 50%	150	584	Cases-48.94 (7.05) Controls 37.79 (7.43)	Cases-Male-% Controls-Male-%	11.7 (5.8)	8.97 (4.71)	p < 0.05
Dogra RK et al. [26]	India	Case–control	ACS	184	350	Cases-36.4 (4.5) Controls-31.1 (6.0)	Cases-Male-96.2% Controls-Male-75.7%	24 (23.9)	27.2 (25.2)	*p* = 0.96
Eftychiou C et al. [76]	Cyprus	Case–control	ACS	63	54	Cases-43.4 (6.0) Controls-58.0 (7.4)	Only male participants	14.5 (5.6)	12.3 (4.1)	*p* = 0.017
Genest JJ et al. [52]	US	Case–control	CAD ≥ 50%	170	255	Cases-50.0 (7.0) Controls-49.0 (6.0)	Only male participants	13.66 (6.44)	10.93 (4.92)	*p* = 0.001
Ghazouani L et al. [42]	Tunisia	Case–control	CAD ≥ 50%	352	390	Cases-58.0 (11.5) Controls-57.3 (7.6)	Cases-Male-76.14% Controls-Male-72.56%	14.8 (5.1)	14 (6.9)	*p* = 0.200
Giles WH et al. [53]	US	Cross-sectional	ACS	259	2914	Cases-68.2 Controls-55.8	Cases-Male-52.10% Controls-Male-47.40%	11.9 (6.44)	10.2 (10.8)	p < 0001
Gokkusu C et al. [36]	Turkey	Case–control	ACS	102	90	Cases-57.1 (10.7) Controls-52.1 (9.7)	Not Mentioned	21.83 (11.1)	9.48 (3.3)	p < 0.001
Golbahar J [63]	Iran	Case–control	CAD ≥ 50%	195	201	Cases-59.6 (11.3) Controls-52.9 (10.2)	Only male participants	11.4 (4.9)	8.4 (3.1)	p < 0.001
Gupta M et al. [27]	India	Case–control	CAD	100	50	Not mentioned	Not mentioned	16.57 (6.86)	11.47 (5.19)	p < 0.001
Gupta MD et al. [28]	India	Case–control	ACS	125	103	Cases-29.33 (4.01) Controls-27.97 (4.07)	Cases-Male-95.0% Controls-Male-58.0%	36.23 (29.18)	30.08 (24.46)	*p* = 0.826
Gupta SK et al. [29]	India	Case–control	CAD	199	200	Cases-37.4 (5.8) Controls-36.35 (6.58)	Cases-Male-92.96% Controls-Male-92.0%	22.14 (10.62)	17.38 (8.46)	p < 0.001
Huh HJ et al. [69]	South Korea	Cross-sectional	CAD	163	50	Average age of cases & controls-60 years; range – 2 to 80 years	150 male and 63 females. Proportion of male among cases and control not mentioned separately	12 (2.5)	12.2 (3.3)	p > 0.05
Iqbal MP et al. [48]	Pakistan	Case–control	ACS	224	126	Cases-52.83 (9.12) Controls-51.23 (7.95)	Cases-Male-75.4% Controls-Male-75.4%	18 (8.36)	16.42 (4.94)	p < 0.006
Iqbal MP et al. [49]	Pakistan	Case–control	ACS	203	205	Cases-41.6 (4.5) Controls-41.3 (4.7)	Cases-Male-71.9% Controls-Male-72.2%	23.2 (17.4)	23.45 (18.6)	*p* = 0.78
Jayarajan K et al. [30]	India	Case–control	ACS	28	50	Cases-44.04 (9.59) Controls-41.04 (9.61)	Not mentioned	21.93 (10.505)	14.34 (4.796)	*p* = 0.001
Jemaa R et al. [43]	Tunisia	Cross-sectional	ACS	310	250	Cases-54.2 (8.6) Controls-51.1 (9.2)	Only male participants	14.9 (8.3)	14.1 (4.9)	*p* = 0.493
Kawashiri M et al. [73]	Japan	Case–control	CAD ≥ 50%	57	138	Cases-53.0 (8.0) Controls-52.0 (8.0)	Only male participants	13.4 (7)	10.6 (3)	*p* = 0.0002
Kazemi MB et al. [64]	Iran	Cross-sectional	CAD	133	64	Not Mentioned	Not Mentioned	16.98 (6.49)	13.47 (5.67)	p < 0.0001
Kerkeni M et al. [44]	Tunisia	Case–control	CAD	100	120	Cases-59.0 (10.0) Controls-54.0 (10.0)	Cases-Male-74.0% Controls-Male-72.5%	15.86 (8.63)	11.9 (3.25)	p < 0.001
Li S et al. [60]	China	Case–control	CAD ≥ 50%	170	105	Cases-56.1 (6.24) Controls-55.3 (6.83)	Cases-Male-54.12% Controls-Male-53.33%	22.63 (5.18)	8.11 (2.42)	p < 0.05
Lin PT et al. [59]	Taiwan	Case–control	CAD ≥ 50%	121	155	Cases-59.1 (8.43) Controls-58.8 (7.08)	Cases-Male-63.6% Controls-Male-54.8%	10.7 (5.6)	9.6 (2.2)	*p* = 0.799
Loehrer FM et al. [70]	Switzerland	Case–control	CAD	68	45	Cases-52.0 (12.0) Controls-44.0 (11.0)	Cases-Male-80.0% Controls-Male-51.1%	10.7 (4.1)	7.7 (2.3)	p < 0.001
Martin NJ et al. [54]	US	Case–control	CAD	66	43	Cases-64.0 (8.10) Controls-56.5 (12.68)	Cases-Male-84.0% Controls-Male-73.0%	9.66 (3.35)	7.81 (2.5)	*p* = 0.003
Montalescot G. et al. [72]	France	Case–control	CAD	50	50	Cases-56.0 (2.0) Controls-55.0 (2.0)	Cases-Male-84.0% Controls-Male-84.0%	11.7 (4.949)	9.9 (3.535)	*p* = 0.03
Muzaffar R. et al. [50]	Pakistan	Cross-sectional	CAD	105	105	Cases-44.7 (8.6) Controls-43 (8.4)	Cases-Male-75.2% Controls-Male-76.2%	22.33 (9.22)	12.59 (3.73)	p < 0.0001
Noichri Y et al. [45]	Tunisia	Case–control	ACS	108	81	Cases-63.0 (12.0) Controls-59.0 (9.0)	Cases-Male-70.0% Controls-Male-38.0%	26.9 (15.47)	14.75 (2.69)	p < 0.001
Oudi ME et al. [46]	Tunisia	Case–control	ACS	122	80	Cases-63.86 (10.07) Controls-57.02 (4.32)	Cases-Male-63.11% Controls-Male-53.75%	17.67 (8.32)	13.95 (6.09)	p < 0.01
Ozkan Y et al. [37]	Turkey	Case–control	CAD	50	23	Cases-58.7 (11.5) Controls-50.3 (6.1)	Cases-Male-80.0% Controls-Male-35.0%	15.9 (4.8)	7.7 (1.9)	p < 0.0001
Palazhy S et al. [31]	India	Cross-sectional	CAD	151	84	Cases-56.7 Controls-50.1	Only male participants	16.6 (8.6)	13.6 (5.6)	*p* = 0.008
Puri A et al. [22]	India	Case–control	CAD	51	15	Cases-41.37 (4.48) Controls-41.93 (2.72)	Cases-Male-82.35% Controls-Male-80.0%	27.8 (13.11)	13.22 (7.36)	*p* = 0.0001
Rallidis LS et al. [77]	Greece	Case–control	ACS	144	103	Cases-32.3 (3.3) Controls-31.8 (3.1)	85.4% patients were male Proportion of male among control not mentioned	13.9 (8.6)	11.8 (4.9)	*p* = 0.02
Rothenbacher D et al. [75]	Germany	Case–control	CAD ≥ 50%	312	479	Cases-57.7 (7.4) Controls-55.8 (7.2)	Cases-Male-85.58% Controls-Male-74.95%	9.43 (3.63)	8.91 (3.07)	*p* = 0.145
Shah H et al. [51]	Pakistan	Case–control	CAD	128	30	Cases-33.81 (2.74) Controls-32.84 (2.14)	Cases-Male-67.20% Controls-Male-68.10%	44.5 (14.01)	6.3 (2.05)	p < 0.0001
Shenoy V et al. [23]	India	Cross-sectional	CAD	51	19	Cases-54 (7.47) Controls-Not mentioned	Cases-Male-82.35% Controls-Male-47.36%	23.35 (6.08)	13.68 (6.27)	p < 0.001
Stampfer MJ et al. [55]	US	Nested case–control	ACS	271	271	Cases-58.9 (8.5) Controls-58.9 (8.6)	Only male participants	11.1 (4)	10.5 (2.8)	*p* = 0.026
Sugijo H et al. [68]	Indonesia	Cross-sectional	CAD	30	30	Cases-44.6 (8.94) Controls-48.83 (8.76)	Cases-Male-86.7% Controls-Male-76.7%	13.91 (4.55)	10.97 (3.45)	*p* = 0.004
Szczeklik A et al. [71]	Poland	Case–control	CAD ≥ 50%	161	211	Cases-43.6 (4.7) Controls-43.7 (5.6)	Only male participants	16.7 (10.8)	13.2 (3.7)	p > 0.05
Wu DF et al. [61]	China	Case–control	CAD ≥ 50%	872	774	Cases-63.27 (10.4) Controls-63.66 (29.81)	Cases-Male-72.71% Controls-Male-55.81%	15.11 (5.2)	13.5 (5.84)	p < 0.001
Yildirir A et al. [38]	Turkey	Cross-sectional	CAD ≥ 50%	58	25	Cases-56.0 (11.0) Controls-53.0 (10.0)	Cases-Male-67.24% Controls-Male-40.0%	15 (5.7)	13.5 (5.6)	p > 0.05
Yilmaz H et al. [39]	Turkey	Case–control	CAD ≥ 50%	79	93	Cases-55.9 (11.3) Controls-54.6 (12.1)	Cases-Male-70.88% Controls-Male-69.89%	13.9 (7.4)	11.8 (5.2)	p > 0.05
Zhang SY et [62]	China	Case–control	CAD	875	956	Cases-46.20 (4.32) Controls-43.96 (5.52)	Cases-Male-91.20% Controls-Male-90.27%	18.85 (6.93)	13.56 (5.83)	p < 0.001

### Risk of bias assessment

Upon assessing the risk of bias, it was determined that out of the 59 studies included in the meta-analysis, 55 were classified as being of good or fair quality, while four studies were of poor quality (Additional file 1: Table S4).

### Outcomes

**Fig. 2:**
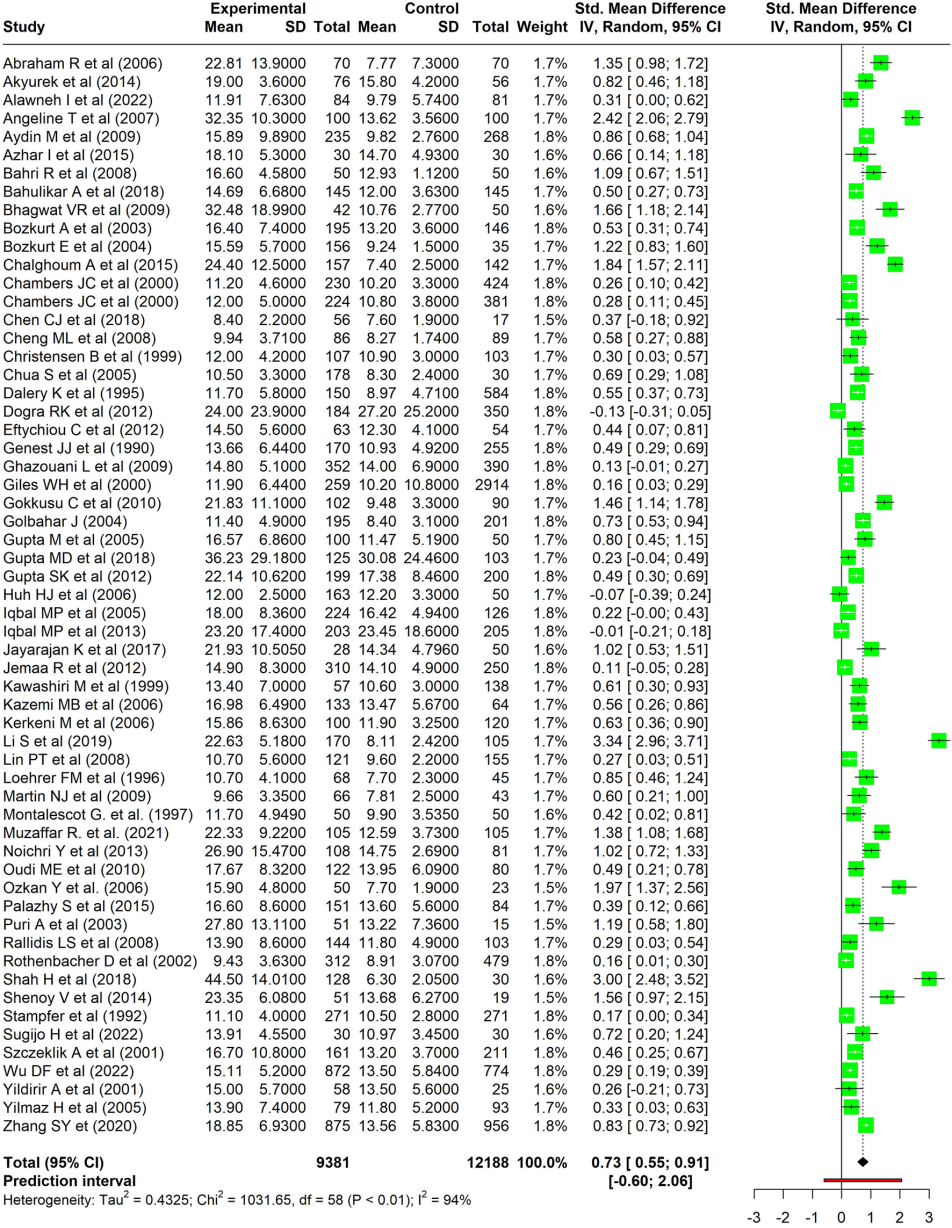
Forest plot showing the pooled estimate of the standard mean difference in the homocysteine levels between CAD and controls

The pooled SMD of the homocysteine levels between the cases and controls was 0.73 (95% CI 0.55–0.91), which implies a significantly higher homocysteine levels among the cases than the controls. There was high heterogeneity between the studies (I^2^ = 94%, p < 0.01). The prediction interval was between − 0.60 and 2.06 (Fig. 2). Sensitivity analysis following the removal of poor-quality studies resulted in a similar pooled SMD of 0.73 (95% CI 0.54–0.91) (Additional file 2: Figure S1).

Two of the 59 studies were identified as potential outliers (Additional file 2: Figure S2), and the sensitivity analysis revealed a slightly lower SMD of 0.64 (95% CI 0.50–0.78). Although there was a minor decrease in heterogeneity, it still remained at a high level with an I^2^ value of 92% (p < 0.01). Additionally, the prediction interval became narrower, ranging from − 0.35 to 1.63 (Additional file 2: Figure S3).

The leave-one-out analysis revealed that there was not much variation observed in the heterogeneity, with I^2^ values ranging from 93 to 94%. Similarly, the pooled standardized mean difference (SMD) estimate remained relatively stable, varying from 0.68 to 0.75 throughout the analysis (Additional file 2: Figure S4).

**Fig. 3:**
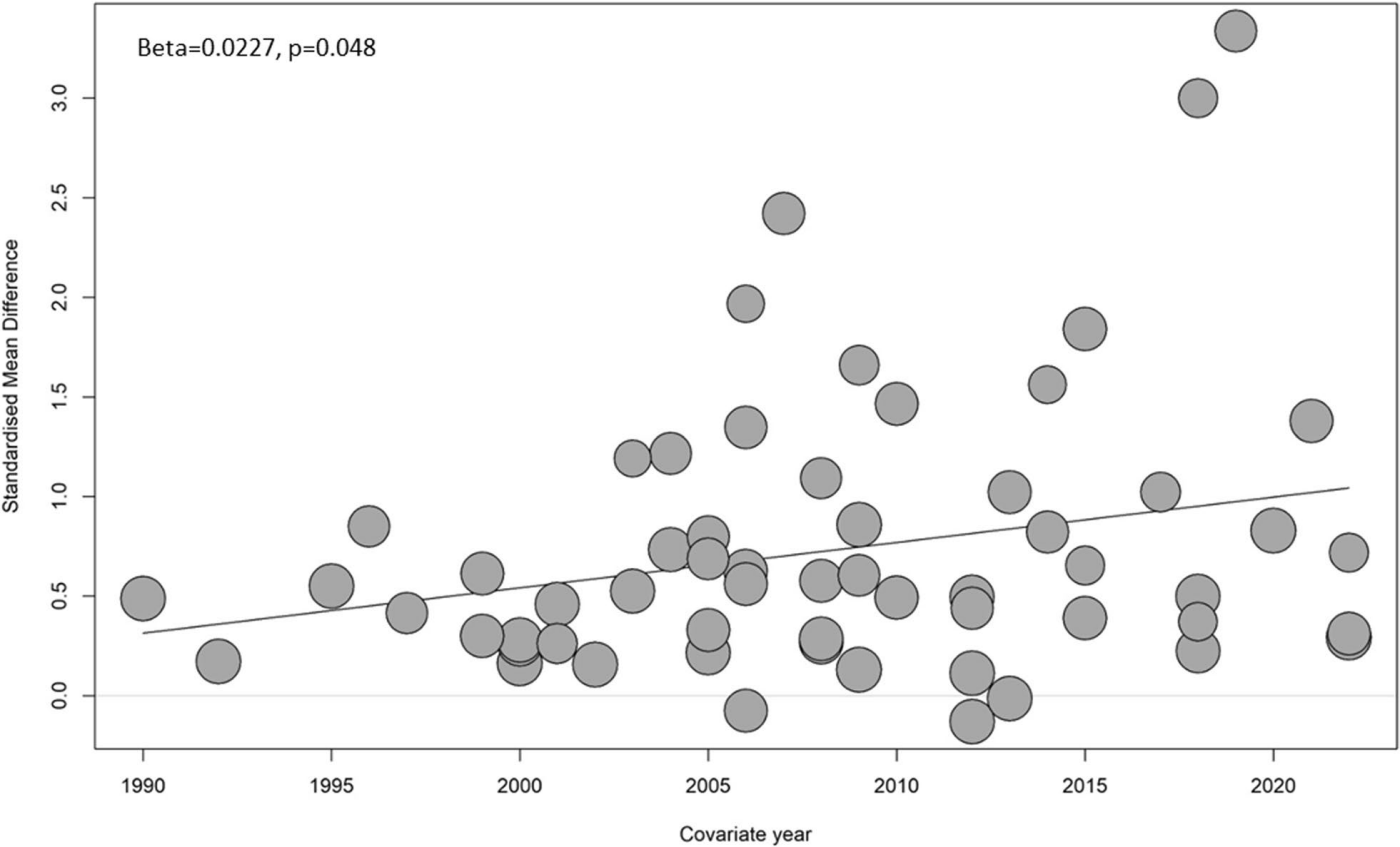
Meta-regression of the outcome measure over the time

Table 2 presents the pooled estimate resulting from the subgroup analysis. Except for the Europe subgroup (I^2^ 51%), heterogeneity remained substantial and even among all the subgroup analysis. Diagnosis (ACS/CAD) and the fasting status of the blood sample did not significantly affect the SMD between the cases and controls. There was a significant difference in the homocysteine levels association with the CAD/ACS between different regions of the world, with the highest SMD among the Asian studies (0.85 [95% CI 0.60–1.10]), while the European studies reported the lowest SMD between the cases and controls (0.32 [95% CI 0.18–0.46]). Similarly, the time period of the study had a significant impact on the pooled SMD of the Hcy levels, with the post-2000 SMDs being significantly higher than the pre-2001 levels. Meta-regression also revealed that the strength of association was increasing over the period in the index meta-analysis (Beta = 0.0227, *p* = 0.048).(Fig. 3). Relatively, studies that included only males had a significantly lower SMD in the homocysteine levels (0.40 [95% CI 0.28;0.53]) than the studies where both genders were included (0.81 [95% CI 0.59–1.03]) (Table 2).

**Table 2:** Pooled estimates of Hcy SMD according to the subgroup analysis

Subgroup	No. of studies	Pooled estimate (95% CI)	I^2^	*p* value
Case group				0.52
ACS	30	0.72 (0.48–0.96)	94%	
CAD (> = 50% occlusion)	16	0.60 (0.21–1.00)	95%	
CAD (criteria unspecified)	13	0.92 (0.47–1.36)	92%	
Region				< 0.01
Asia	39	0.85 (0.60–1.10)	95%	
Europe	8	0.32 (0.18–0.46)	51%	
America	5	0.37 (0.11–0.63)	80%	
Africa	7	0.75 (0.18–1.32)	96%	
Gender				< 0.01
Male & Females	49	0.81 (0.59–1.03)	95%	
Males only	10	0.40 (0.28–0.53)	70%	
Type of blood sample				0.75
Fasting	49	0.75 (0.54–0.96)	95%	
Non-fasting	3	0.49 (− 1.00–1.98)	93%	
Not reported	7	0.69 (0.27–1.10)	87%	
Time of study				< 0.01
After 2020	4	0.67 (− 0.16–1.49)	94%	
2011–2020	18	0.90 (0.43–1.38)	97%	
2001–2010	27	0.75 (0.52–0.99)	92%	
Before 2001	10	0.37 (0.23–0.52)	69%

**Fig. 4:**
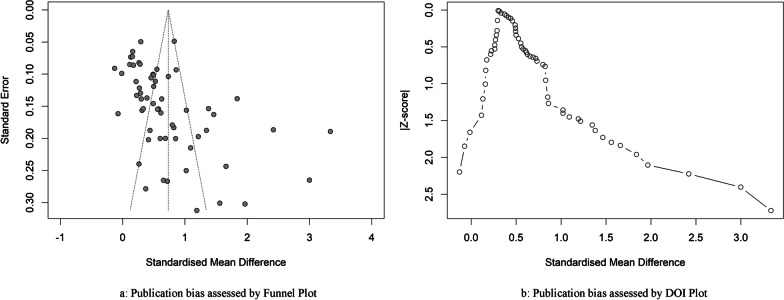
**a** Publication bias assessed by funnel plot and **b** publication bias assessed by Doi Plot

Funnel plot and Doi plot asymmetry analysis indicated the presence of potential publication bias. This bias was statistically represented by the LFK index, which yielded a value of 2.69 (Fig. 4a, b).

The certainty of the evidence was rated low, owing to the observational nature of the studies, high heterogeneity, and publication bias (Table 3).

**Table 3:** Certainty of evidence on the association between the homocysteine and CAD by GRADE profile

No. of studies	Risk of bias	Inconsistency	Indirectness	Imprecision	Publication bias	Summary findings			Certainty of evidence
						Casesn	Controln	SMD (95% CI)	
59	No serious limitations	Serious limitations	No serious limitations	No serious imprecision	Serious limitations	9381	12,188	0.73 (0.55–0.91)	⊕ ⊕ OO LOW

## Discussion

Of the novel biomarkers of CAD, plasma homocysteine is being studied to determine whether it is an independent modifiable risk factor or not. The mechanism by which high concentrations of plasma homocysteine lead to atherothrombosis is endothelial cell damage impairing its function, which has been shown in many in vitro studies as well. High Hcy levels increase oxidative stress, inducing inflammatory processes, increasing endoplasmic reticulum stress and apoptosis, increasing autoimmune reactions, and ultimately increasing the coagulation cascade [68, 78]. The index meta-analysis, which is the first to present the pooled estimate of this association, included a total of 9381 cases of CAD and 12,188 controls from the 59 studies and revealed a significantly higher plasma homocysteine concentration among the cases (SMD of 0.73, 95% CI: 0.55–0.91). This suggests a significant association of homocysteine with the occurrence of CAD, although the overall certainty of the findings is low, mostly due to high heterogeneity. The high heterogeneity in the association reported in the index meta-analysis could be due to the difference in inclusion and exclusion criteria of cases and controls, inadequate sample size, different methods of homocysteine measurement and fasting status of blood sample collection, the presence of other traditional risk factors, and the wide range of time periods of the included studies. Plasma homocysteine concentration is affected by many factors like age, gender, ethnicity, nutritional deficiencies like folate and Vitamin B12, and renal and liver function [79]. In addition to these factors, the enzyme methylene tetrahydrofolate reductase, encoded by the MTHFR gene, also regulates the homocysteine level. This gene metabolizes and removes homocysteine by using folate. A polymorphism of the gene MTHFR C677T reduces the efficiency of the enzyme and leads to an increase in plasma Hcy concentration [80], which might have also contributed to the heterogeneity. Twenty-one of the 59 studies have investigated the different genetic and environmental interactions involved in the occurrence of CAD. Girelli D. et al. concluded in their study that the MTHFR C677T mutation was not associated with CAD, but genetic–environmental interaction might contribute to the vascular risk by raising Hcy, which is why the folate level is low [81]. Similar findings were produced by Huh HJ et al. who found that gene–nutrient interactions can increase the risk for CAD based on specific threshold folate levels [69].

Studies adopted different case groups, like patients with acute conditions or those who had coronary events in the past, where the diagnosis was based on the extent of stenosis on angiography. The pooled estimate of SMD was high among the CAD group, where patients with any degree of stenosis were included. Heterogeneity was little reduced (from 95% in the CAD group with ≥50% stenosis, 94% in the ACS group and 92% in the CAD-criteria unspecified group). Various studies reported that patients with severe CAD in terms of the number of vessels affected or higher Gensini scores had significantly elevated mean total homocysteine levels [23, 82]. But Bozkurt A et al. reported that homocysteine concentration was unrelated to the extent (in terms of the number of vessels affected) and severity of the disease [34]. Pooled SMD is high for the studies done in Asian countries (pooled SMD 0.85, 95% CI: 0.60–1.10), followed by African countries (pooled SMD 0.75, 95% CI: 0.18–1.32). Heterogeneity is also high among studies done in countries on these two continents, while studies in European or American regions showed less heterogeneity as well as a small pooled SMD. Since homocysteine is believed to be determined by nutritional deficiencies like folate and Vitamin B12 as well as ethnicity, the finding of the index subgroup analysis can be explained by studies exploring the folate levels in various geographical regions. A meta-analysis of MTHFR polymorphism with CHD risk done by Clarke R et al. reported folate levels as low in Asian and European un-supplemented populations, intermediate folate levels in the supplemented European population and un-supplemented US and Australian populations, while high folate levels in the supplemented US and Australian populations [80]. Chambers JC et al., in their two parallel case–control studies done among two ethnic groups, European and Indian Asian men residing in Europe for an average of 27 years, reported that fasting Hcy concentrations were high in Indian Asian men compared to European men, and the age-adjusted difference was 6% [83]. Another study conducted to understand the impact of migration on the risk of coronary heart disease revealed that serum homocysteine levels were significantly higher among the Indian participants residing in India compared to Indian-origin participants who migrated to Sandwell, UK. This was consistent with low serum folate levels among Indian participants compared to migrated participants [84]. CAD is one of the major public health concerns across the world and is known for its multifactorial causation. Thus, genetic as well as environmental factors (folate levels) have an interactive effect on the Hcy levels.

Subgroup analysis based on gender showed that pooled SMD was lower in studies that included only male participants compared to studies with both genders. Studies have shown that males had significantly higher Hcy values than females at each age range, which could be a contributing factor to gender differences in developing CAD [34, 85, 86]. The majority of the studies have documented fasting homocysteine estimation through high-performance liquid chromatography, fluorescence polarization immunoassay, or ELISA methods. Subgroup analysis shows a lower but insignificant pooled SMD when homocysteine is estimated from a non-fasting blood sample. The estimated pooled SMD was lower in the studies published before 2001 (0.37, 95% CI: 0.23–0.52), with substantial heterogeneity. Almost all the studies were done in Europe or the American region except one, which was from Japan. After 2010, all the studies were published in Asian or African countries. So, this difference in pooled estimates over the period of time might be due to the region of study. It is also possible that over the span of three decades, lifestyle has changed considerably and all the known risk factors have become more prevalent, thus increasing the vulnerability for coronary artery disease. Food habits and probably the quality of food have also changed over time, so nutritional deficiencies particularly folate and Vitamin B12 deficiencies could have increased, which in turn is associated with a rise in homocysteine levels, particularly in low- and middle-income countries in Asia and Africa.

### Strengths and limitations

The index meta-analysis is the first study to present the global, regional, and temporal pooled association estimates of the Hcy with CAD. The quality of the studies was evaluated by standard tools, and sensitivity analysis was undertaken to improve the robustness of the findings. Heterogeneity was explored by means of appropriate and feasible subgroup analyses. The GRADE profile was applied to present the certainty of the evidence from the meta-analysis. However, the index meta-analysis had the following limitations: High heterogeneity between the studies persisted even after subgroup analysis might be due to the presence of publication bias, language bias or genetic reasons. Although we searched three of the major databases (PubMed, Scopus and Cochrane) along with Google Scholar, more databases could not be included, because of limited resource availability.

## Conclusions

Overall, even though higher homocysteine levels might have a significant association with coronary artery diseases, the certainty of evidence is low. Within the population groups, Asian and African populations showed a greater strength of association than their European and American counterparts. High heterogeneity, which is a major factor impacting the certainty of the evidence, needs to be explored in future studies by reporting and conducting subgroup analysis based on determinants such as genetics, sex and folate levels. Primary studies can be designed to alleviate all probable confounding factors to assess the predictive role of homocysteine in CAD and whether it is a modifiable risk factor.

### Supplementary Information

**Additional file 1. Table S1: **PRISMA Checklist (2020).** Table S2: **Inclusion and exclusion criteria.** Table S3: **The adjusted search terms as per searched electronic databases [as of 17.04.2023].** Table S4: **Quality assessment with the use of National Heart, Lung, and Blood Institute (NHLBI) quality assessment tool

**Additional file 2. Figure S1: I**nfluence diagnostics.** Figure S2: **Forest plot showing the pooled estimates after removing poor-quality studies.** Figure S3: **Forest plot showing the pooled estimates after removing two outlier studies.** Figure S4: **Leave one out analysis

## Data Availability

The datasets used and/or analyzed during the current study are included in the manuscript and supplementary materials.

## Abbreviations

ACS: Acute coronary syndrome
CAD: Coronary artery disease
CHD: Coronary heart disease
CI: Confidence interval
CVD: Cardiovascular disease
Hcy: Homocysteine
NHLBI: National Heart, Lung, and Blood Institute
RR: Risk ratio
SD: Standard deviation
SMD: Standardized mean difference
SRMA: Systematic review and meta-analysis
tPA: Tissue plasminogen activator
