# Supplementary material for: Association between homocysteine and coronary artery disease—trend over time and across the regions: a systematic review and meta-analysis
Source: Egypt Heart J. 2024 Feb 27;76:29. doi: 10.1186/s43044-024-00460-y (PMC10897093; doi:10.1186/s43044-024-00460-y)
Supplement: Supplementary file 1 — Additional file 1. Table S1: PRISMA Checklist (2020). Table S2: Inclusion and exclusion criteria. Table S3: The adjusted search terms as per searched electronic databases [as of 17.04.2023]. Table S4: Quality assessment with the use of National Heart, Lung, and Blood Institute (NHLBI) quality assessment tool [file 43044_2024_460_MOESM1_ESM.docx]

**Table S1: PRISMA Checklist (2020)**

| **Section and Topic** | **Item #** | **Checklist item** | **Location where item is reported** |
| --- | --- | --- | --- |
| **TITLE** | | |  |
| Title | 1 | Identify the report as a systematic review. | Pg 1 |
| **ABSTRACT** | | |  |
| Abstract | 2 | Made as per the Journal guidelines | Pg 1 |
| **INTRODUCTION** | | |  |
| Rationale | 3 | Describe the rationale for the review in the context of existing knowledge. | Pg 2, 3 |
| Objectives | 4 | Provide an explicit statement of the objective(s) or question(s) the review addresses. | Pg 3 |
| **METHODS** | | |  |
| Eligibility criteria | 5 | Specify the inclusion and exclusion criteria for the review and how studies were grouped for the syntheses. | Supplementary Table S2 |
| Information sources | 6 | Specify all databases, registers, websites, organisations, reference lists and other sources searched or consulted to identify studies. Specify the date when each source was last searched or consulted. | Pg 4 |
| Search strategy | 7 | Present the full search strategies for all databases, registers and websites, including any filters and limits used. | Supplementary Table S3 |
| Selection process | 8 | Specify the methods used to decide whether a study met the inclusion criteria of the review, including how many reviewers screened each record and each report retrieved, whether they worked independently, and if applicable, details of automation tools used in the process. | Pg 4-5 |
| Data collection process | 9 | Specify the methods used to collect data from reports, including how many reviewers collected data from each report, whether they worked independently, any processes for obtaining or confirming data from study investigators, and if applicable, details of automation tools used in the process. | Pg 5 |
| Data items | 10a | List and define all outcomes for which data were sought. Specify whether all results that were compatible with each outcome domain in each study were sought (e.g. for all measures, time points, analyses), and if not, the methods used to decide which results to collect. | Pg 5, 6 |
|  | 10b | List and define all other variables for which data were sought (e.g. participant and intervention characteristics, funding sources). Describe any assumptions made about any missing or unclear information. | Pg 5, 6 |
| Study risk of bias assessment | 11 | Specify the methods used to assess risk of bias in the included studies, including details of the tool(s) used, how many reviewers assessed each study and whether they worked independently, and if applicable, details of automation tools used in the process. | Pg 7 |
| Effect measures | 12 | Specify for each outcome the effect measure(s) (e.g. risk ratio, mean difference) used in the synthesis or presentation of results. | Pg 5 |
| Synthesis methods | 13a | Describe the processes used to decide which studies were eligible for each synthesis (e.g. tabulating the study intervention characteristics and comparing against the planned groups for each synthesis (item #5)). | Pg 5, 6 |
|  | 13b | Describe any methods required to prepare the data for presentation or synthesis, such as handling of missing summary statistics, or data conversions. | NA |
|  | 13c | Describe any methods used to tabulate or visually display results of individual studies and syntheses. | Pg 6 |
|  | 13d | Describe any methods used to synthesize results and provide a rationale for the choice(s). If meta-analysis was performed, describe the model(s), method(s) to identify the presence and extent of statistical heterogeneity, and software package(s) used. | Pg 5, 6 |
|  | 13e | Describe any methods used to explore possible causes of heterogeneity among study results (e.g. subgroup analysis, meta-regression). | Pg 5, 6 |
|  | 13f | Describe any sensitivity analyses conducted to assess robustness of the synthesized results. | Pg 6 |
| Reporting bias assessment | 14 | Describe any methods used to assess risk of bias due to missing results in a synthesis (arising from reporting biases). | Pg 7 |
| Certainty assessment | 15 | Describe any methods used to assess certainty (or confidence) in the body of evidence for an outcome. | Pg 6 |
| **RESULTS** | | |  |
| Study selection | 16a | Describe the results of the search and selection process, from the number of records identified in the search to the number of studies included in the review, ideally using a flow diagram. | Figure 1 |
|  | 16b | Cite studies that might appear to meet the inclusion criteria, but which were excluded, and explain why they were excluded. | Pg 8 & Figure 1 |
| Study characteristics | 17 | Cite each included study and present its characteristics. | Pg 7, 8, table 1 |
| Risk of bias in studies | 18 | Present assessments of risk of bias for each included study. | Supplementary Tables S4 |
| Results of individual studies | 19 | For all outcomes, present, for each study: (a) summary statistics for each group (where appropriate) and (b) an effect estimate and its precision (e.g. confidence/credible interval), ideally using structured tables or plots. | Figure 2 |
| Results of syntheses | 20a | For each synthesis, briefly summarise the characteristics and risk of bias among contributing studies. | Table 1, Supplementary Tables S4 |
|  | 20b | Present results of all statistical syntheses conducted. If meta-analysis was done, present for each the summary estimate and its precision (e.g. confidence/credible interval) and measures of statistical heterogeneity. If comparing groups, describe the direction of the effect. | Figure 2 |
|  | 20c | Present results of all investigations of possible causes of heterogeneity among study results. | Pg – 8-9 |
|  | 20d | Present results of all sensitivity analyses conducted to assess the robustness of the synthesized results. | Pg 7-9, Supplementary figure S2, S3 |
| Reporting biases | 21 | Present assessments of risk of bias due to missing results (arising from reporting biases) for each synthesis assessed. | Pg 7, Figure 4a & 4b |
| Certainty of evidence | 22 | Present assessments of certainty (or confidence) in the body of evidence for each outcome assessed. | Pg 8, Table 3 |
| **DISCUSSION** | | |  |
| Discussion | 23a | Provide a general interpretation of the results in the context of other evidence. | Pg 10-13 |
|  | 23b | Discuss any limitations of the evidence included in the review. | Pg 12-15 |
|  | 23c | Discuss any limitations of the review processes used. | Pg 13 |
|  | 23d | Discuss implications of the results for practice, policy, and future research. | Pg 13 |
| **OTHER INFORMATION** | | |  |
| Registration and protocol | 24a | Provide registration information for the review, including register name and registration number, or state that the review was not registered. | Pg 6 |
|  | 24b | Indicate where the review protocol can be accessed, or state that a protocol was not prepared. | Pg 6 |
|  | 24c | Describe and explain any amendments to information provided at registration or in the protocol. | NA |
| Support | 25 | Describe sources of financial or non-financial support for the review, and the role of the funders or sponsors in the review. | In Title page |
| Competing interests | 26 | Declare any competing interests of review authors. | In Title page |
| Availability of data, code and other materials | 27 | Report which of the following are publicly available and where they can be found: template data collection forms; data extracted from included studies; data used for all analyses; analytic code; any other materials used in the review. | Pg 33-48 |

**Table S2 Inclusion and exclusion criteria**

**Research Question:** What is the association between the plasma homocysteine level and the occurrence of coronary artery disease?

| **Inclusion** | | **Exclusion** |
| --- | --- | --- |
| **Participants / Population** | Studies were included if Case group represents patients of Coronary Artery Disease including Acute Coronary  Syndrome diagnosed by ECG changes or elevated cardiac enzymes or coronary angiography showing any degree of stenosis  Studies were Control group represents participants free from Coronary Artery Disease confirmed by ECG / estimation of cardiac enzymes / coronary angiography / exercise tolerance test / history   - All gender - All age groups - All the countries | Studies were excluded if   - Case group had Stable Angina Pectoris patients without diagnosis through coronary angiography - Case group represents different outcome like death due to CAD or recurrence of disease - Control group had subjects with coronary angiography showing any stenosis |
| **Disease** | Coronary Artery Disease (including Acute Coronary Syndrome) | Studies have been excluded if cases are selected from any other disease like diabetes or other vascular disease |
| **Outcome** | Association between plasma homocysteine level and occurrence of coronary artery disease (measured by mean difference in plasma homocysteine among case and control group) | Studies have been excluded if plasma homocysteine values are not reported as arithmetic mean and standard deviation |
| **Study Designs** | Observational studies like cross sectional studies, case control studies and nested case control studies | Case reports, case series, review articles and cohort studies |
|  | Geography-Global level  Date of Search- Publish till December 31^st^ December 2022  English Language  Human studies |  |
|  | Published and Un-published data |  |

**Table S3. The adjusted search terms as per searched electronic databases [as of 17.04.2023]**

| Database | No | Search Query | Results |
| --- | --- | --- | --- |
| Cochrane | | | |
|  | #1 | ("acute coronary syndrome"):ti,ab,kw OR (ACS):ti,ab,kw OR ("acute myocardial infarction"):ti,ab,kw OR (STEMI):ti,ab,kw OR (NSTEMI):ti,ab,kw OR ("unstable angina"):ti,ab,kw OR ("coronary artery disease"):ti,ab,kw OR (CAD):ti,ab,kw AND ("coronary heart disease"):ti,ab,kw AND (CHD):ti,ab,kw | 40,747 |
|  | #2 | (hyperhomocysteinemia):ti,ab,kw OR (homocysteinemia):ti,ab,kw OR (homocystein):ti,ab,kw | 3205 |
|  | #3 | #1 AND #2 | 230 |
| PubMed | | | |
|  | #1 | **((((((((("acute coronary syndrome"[Title/Abstract]) OR (ACS[Title/Abstract])) OR ("acute myocardial infarction"[Title/Abstract])) OR (STEMI[Title/Abstract])) OR (NSTEMI[Title/Abstract])) OR ("unstable angina"[Title/Abstract])) OR ("coronary artery disease"[Title/Abstract])) OR (CAD[Title/Abstract])) OR ("coronary heart disease"[Title/Abstract])) OR (CHD[Title/Abstract])** | 297,929 |
|  | #2 | **((hyperhomocysteinemia[Title/Abstract]) OR (homocysteinemia[Title/Abstract])) OR (homocysteine[Title/Abstract])** | 26,193 |
|  | #3 | #1 AND #2 AND AND (humans[Filter]) AND (english[Filter])) | 1608 |
| Scopus | | | |
|  | #1 | (((((((((TITLE-ABS-KEY(“acute coronary syndrome”)) OR (TITLE-ABS-KEY(ACS))) OR (TITLE-ABS-KEY(“acute myocardial infarction”)))) OR (TITLE-ABS-KEY(STEMI))))) OR (TITLE-ABS-KEY(NSTEMI)))))) OR (TITLE-ABS-KEY(“unstable angina”))))))) OR (TITLE-ABS-KEY(“coronary artery disease”)))))))) OR (TITLE-ABS-KEY(CAD))))))))) OR (TITLE-ABS-KEY(“coronary heart disease”)))))))))) OR (TITLE-ABS-KEY(CHD)) | 602,088 |
|  | #2 | **((TITLE-ABS-KEY(hyperhomocysteinaemia)) OR (TITLE-ABS-KEY(homocysteinemia))) OR (TITLE-ABS-KEY(homocysteine))** | 42,888 |
|  | #3 | #1 AND #2 | 1999 |

**Table S4: Quality assessment with the use of National Heart, Lung, and Blood Institute (NHLBI) quality assessment tool**

| Author | Q.1 | Q.2 | Q.3 | Q.4 | Q.5 | Q.6 | Q.7 | Q.8 | Q.9 | Q.10 | Q.11 | Q.12 | Overall Quality |
| --- | --- | --- | --- | --- | --- | --- | --- | --- | --- | --- | --- | --- | --- |
| Abraham R et al (2006) | Yes | Yes | No | Yes | Yes | Yes | NA | Yes | No | Yes | NR | No | Fair |
| Akyurek et al (2014) | Yes | Yes | No | Yes | Yes | Yes | NR | Yes | No | Yes | NR | No | Fair |
| Alawneh I et al (2022) | Yes | Yes | Yes | Yes | Yes | Yes | NR | Yes | No | Yes | NR | No | Fair |
| Angeline T et al (2007) | Yes | CD | No | Yes | Yes | Yes | NA | Yes | No | Yes | NR | No | Fair |
| Aydin M et al (2009) | Yes | Yes | No | Yes | Yes | Yes | Yes | Yes | No | Yes | NR | Yes | Good |
| Azhar I et al (2015) | Yes | Yes | No | Yes | Yes | Yes | NR | Yes | No | Yes | NR | No | Fair |
| Bahri R et al (2008) | Yes | Yes | No | Yes | Yes | Yes | Yes | Yes | No | Yes | NR | No | Fair |
| Bahulikar A et al (2018) | Yes | Yes | Yes | Yes | Yes | Yes | NA | Yes | No | Yes | NR | No | Fair |
| Bhagwat VR et al (2009) | Yes | Yes | No | Yes | Yes | Yes | Yes | Yes | No | Yes | NR | No | Fair |
| Bozkurt A et al (2003) | Yes | Yes | No | Yes | Yes | Yes | NA | Yes | No | Yes | NR | Yes | Fair |
| Bozkurt E et al (2004) | Yes | Yes | No | Yes | Yes | Yes | NA | CD | No | Yes | Yes | No | Fair |
| Chalghoum A et al (2015) | Yes | Yes | No | Yes | NR | CD | NA | Yes | No | Yes | NR | No | Poor |
| Chambers JC et al (2000) | Yes | Yes | No | Yes | Yes | Yes | NA | Yes | No | Yes | Yes | Yes | Good |
| Chambers JC et al (2000) | Yes | Yes | No | Yes | Yes | Yes | NA | Yes | No | Yes | Yes | Yes | Good |
| Chen CJ et al (2018) | Yes | Yes | No | Yes | CD | CD | NA | Yes | No | CD | NR | No | Poor |
| Cheng ML et al (2008) | Yes | Yes | No | Yes | Yes | Yes | NA | Yes | No | Yes | NR | Yes | Fair |
| Christensen B et al (1999) | Yes | Yes | No | Yes | Yes | Yes | NR | Yes | No | Yes | NR | Yes | Fair |
| Chua S et al (2005) | Yes | CD | No | Yes | Yes | Yes | NA | Yes | No | Yes | NR | No | Fair |
| Dalery K et al (1995) | Yes | Yes | No | CD | Yes | Yes | NA | Yes | No | Yes | NR | No | Fair |
| Dogra RK et al (2012) | Yes | Yes | Yes | Yes | Yes | Yes | Yes | Yes | No | Yes | NR | Yes | Good |
| Eftychiou C et al (2012) | Yes | Yes | Yes | Yes | Yes | Yes | NA | Yes | No | Yes | NR | Yes | Good |
| Genest JJ et al (1990) | Yes | Yes | No | Yes | Yes | Yes | NR | CD | No | Yes | Yes | No | Fair |
| Ghazouani L et al (2009) | Yes | Yes | No | Yes | Yes | Yes | NA | Yes | No | Yes | NR | Yes | Fair |
| Giles WH et al (2000) | Yes | Yes | No | Yes | Yes | Yes | NR | Yes | No | Yes | NR | Yes | Fair |
| Gokkusu C et al (2010) | Yes | Yes | No | Yes | Yes | Yes | NA | Yes | No | Yes | NR | No | Fair |
| Golbahar J (2004) | Yes | Yes | No | Yes | Yes | Yes | Yes | Yes | No | Yes | NR | Yes | Good |
| Gupta M et al (2005) | Yes | Yes | No | Yes | Yes | Yes | NA | Yes | No | Yes | NR | Yes | Fair |
| Gupta MD et al (2018) | Yes | Yes | Yes | Yes | Yes | Yes | NA | Yes | No | Yes | NR | Yes | Good |
| Gupta SK et al (2012) | Yes | Yes | Yes | Yes | Yes | Yes | NA | Yes | No | Yes | NR | Yes | Good |
| Huh HJ et al (2006) | Yes | No | No | Yes | CD | Yes | NA | Yes | No | Yes | No | No | Poor |
| Iqbal MP et al (2005) | Yes | Yes | No | Yes | Yes | Yes | NA | Yes | No | Yes | NR | No | Fair |
| Iqbal MP et al (2013) | Yes | Yes | No | Yes | Yes | Yes | NA | Yes | No | Yes | NR | No | Fair |
| Jayarajan K et al (2017) | Yes | Yes | No | Yes | Yes | CD | NA | Yes | No | CD | NR | No | Poor |
| Jemaa R et al (2012) | Yes | Yes | No | Yes | Yes | Yes | NA | Yes | No | Yes | NR | Yes | Fair |
| Kawashiri M et al (1999) | Yes | CD | No | Yes | Yes | Yes | NA | Yes | No | Yes | Yes | No | Fair |
| Kazemi MB et al (2006) | Yes | Yes | No | Yes | Yes | Yes | NA | Yes | No | Yes | Yes | Yes | Good |
| Kerkeni M et al (2006) | Yes | Yes | No | Yes | Yes | Yes | NA | Yes | No | Yes | Yes | No | Fair |
| Li S et al (2019) | Yes | Yes | No | Yes | Yes | Yes | NR | Yes | No | Yes | NR | No | Fair |
| Lin PT et al (2008) | Yes | Yes | No | Yes | Yes | Yes | NR | Yes | No | Yes | NR | Yes | Fair |
| Loehrer FM et al (1996) | Yes | Yes | No | Yes | Yes | Yes | Yes | Yes | No | Yes | NR | Yes | Good |
| Martin NJ et al (2009) | Yes | Yes | No | Yes | Yes | Yes | Yes | Yes | No | Yes | NR | No | Fair |
| Montalescot G. et al. (1997) | Yes | Yes | No | Yes | Yes | Yes | NR | CD | No | Yes | Yes | No | Fair |
| Muzaffar R et al (2021) | Yes | Yes | No | Yes | Yes | Yes | NR | Yes | No | Yes | NR | Yes | Fair |
| Noichri Y et al (2013) | Yes | Yes | No | Yes | Yes | Yes | NA | Yes | No | Yes | Yes | No | Fair |
| Oudi ME et al (2010) | Yes | Yes | No | Yes | Yes | Yes | NA | Yes | No | Yes | NR | No | Fair |
| Ozkan Y et al. (2006) | Yes | Yes | No | Yes | Yes | Yes | NA | Yes | No | Yes | NR | Yes | Fair |
| Palazhy S et al (2015) | Yes | Yes | No | Yes | Yes | Yes | NA | Yes | No | Yes | NR | No | Fair |
| Puri A et al (2003) | Yes | Yes | No | Yes | Yes | Yes | NR | Yes | No | Yes | NR | Yes | Fair |
| Rallidis LS et al (2008) | Yes | Yes | No | Yes | Yes | Yes | NA | Yes | No | Yes | NR | No | Fair |
| Rothenbacher D et al (2002) | Yes | Yes | No | Yes | Yes | Yes | No | Yes | No | Yes | Yes | Yes | Good |
| Shah H et al (2018) | Yes | Yes | No | Yes | Yes | Yes | Yes | Yes | No | Yes | NR | No | Fair |
| Shenoy V et al (2014) | Yes | Yes | No | Yes | Yes | Yes | NA | Yes | No | Yes | NR | No | Fair |
| Stampfer et al (1992) | Yes | Yes | CD | Yes | Yes | Yes | Yes | Yes | No | Yes | Yes | Yes | Good |
| Sugijo H et al (2022) | Yes | Yes | Yes | Yes | Yes | Yes | NA | Yes | No | Yes | NR | Yes | Good |
| Szczeklik A et al (2001) | Yes | CD | No | Yes | Yes | Yes | NA | Yes | No | Yes | NR | No | Fair |
| Wu DF et al (2022) | Yes | Yes | No | Yes | Yes | Yes | NA | Yes | No | Yes | NR | No | Fair |
| Yildirir A et al (2001) | Yes | CD | No | Yes | Yes | Yes | NA | Yes | No | Yes | Yes | No | Fair |
| Yilmaz H et al (2005) | Yes | CD | No | Yes | Yes | Yes | NA | Yes | No | Yes | NR | No | Fair |
| Zhang SY et (2020) | Yes | Yes | No | Yes | Yes | Yes | NA | Yes | No | Yes | NR | No | Fair |

| Q.1 | 1. Was the research question or objective in this paper clearly stated and appropriate? |
| --- | --- |
| Q.2 | 2. Was the study population clearly specified and defined? |
| Q.3 | 3. Did the authors include a sample size justification? |
| Q.4 | 4. Were controls selected or recruited from the same or similar population that gave rise to the cases (including the same timeframe)? |
| Q.5 | 5. Were the definitions, inclusion and exclusion criteria, algorithms or processes used to identify or select cases and controls valid, reliable, and implemented consistently across all study participants? |
| Q.6 | 6. Were the cases clearly defined and differentiated from controls? |
| Q.7 | 7. If less than 100 percent of eligible cases and/or controls were selected for the study, were the cases and/or controls randomly selected from those eligible? |
| Q.8 | 8. Was there use of concurrent controls? |
| Q.9 | 9. Were the investigators able to confirm that the exposure/risk occurred prior to the development of the condition or event that defined a participant as a case? |
| Q.10 | 10. Were the measures of exposure/risk clearly defined, valid, reliable, and implemented consistently (including the same time period) across all study participants? |
| Q.11 | 11. Were the assessors of exposure/risk blinded to the case or control status of participants? |
| Q.12 | 12. Were key potential confounding variables measured and adjusted statistically in the analyses? If matching was used, did the investigators account for matching during study analysis? |

CD: Cannot Determine, NR: Not Reported, NA: Not Applicable
