# Supplementary material for: Association between homocysteine and coronary artery disease—trend over time and across the regions: a systematic review and meta-analysis
Source: Egypt Heart J. 2024 Feb 27;76:29. doi: 10.1186/s43044-024-00460-y (PMC10897093; doi:10.1186/s43044-024-00460-y)
Supplement: Supplementary file 2 — Additional file 2. Figure S1: Influence diagnostics. Figure S2: Forest plot showing the pooled estimates after removing poor-quality studies. Figure S3: Forest plot showing the pooled estimates after removing two outlier studies. Figure S4: Leave one out analysis [file 43044_2024_460_MOESM2_ESM.pptx]

## Slide 1
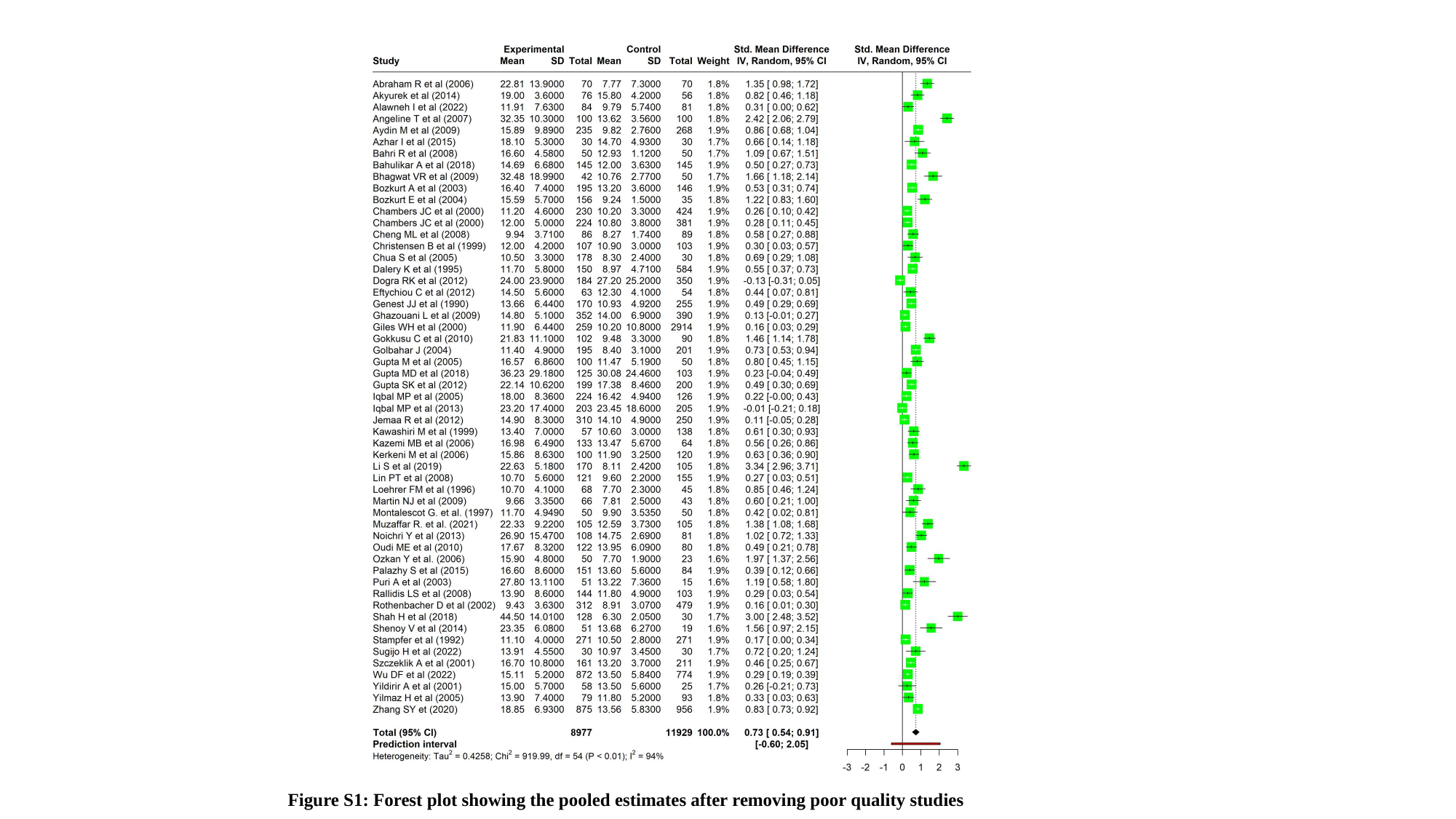

Figure S1: Forest plot showing the pooled estimates after removing poor quality studies

## Slide 2
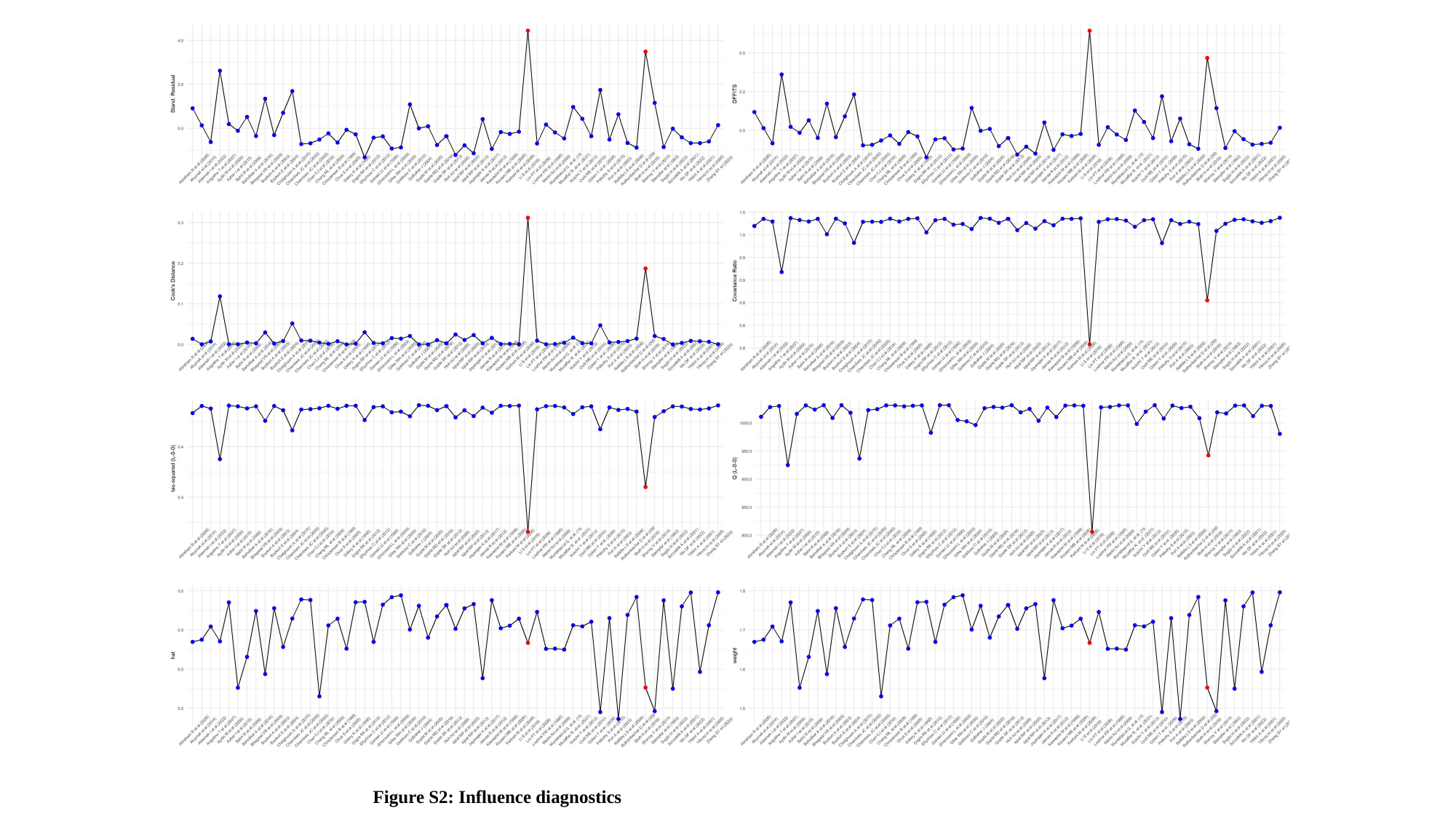

Figure S2: Influence diagnostics

## Slide 3
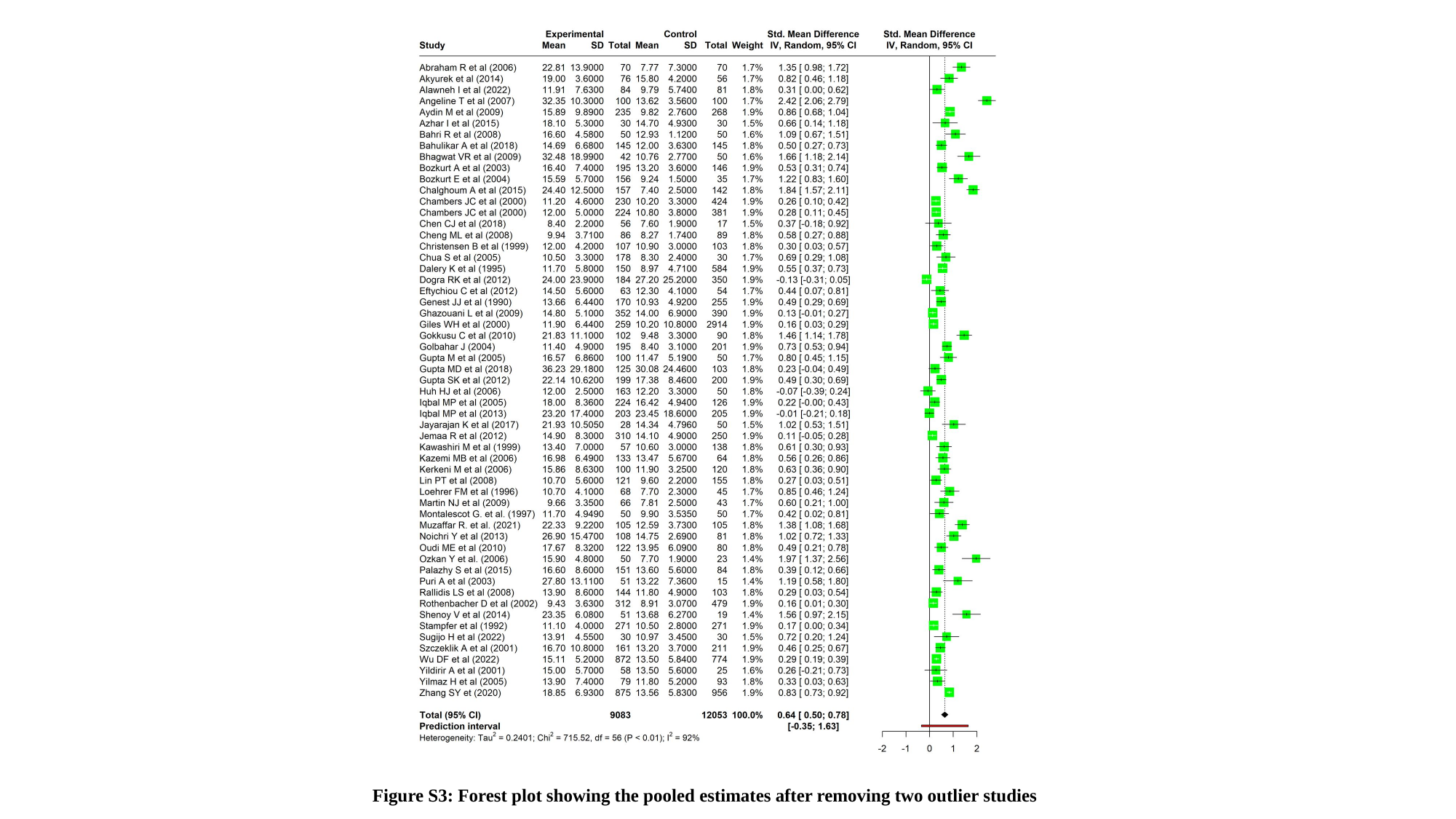

Figure S3: Forest plot showing the pooled estimates after removing two outlier studies

## Slide 4
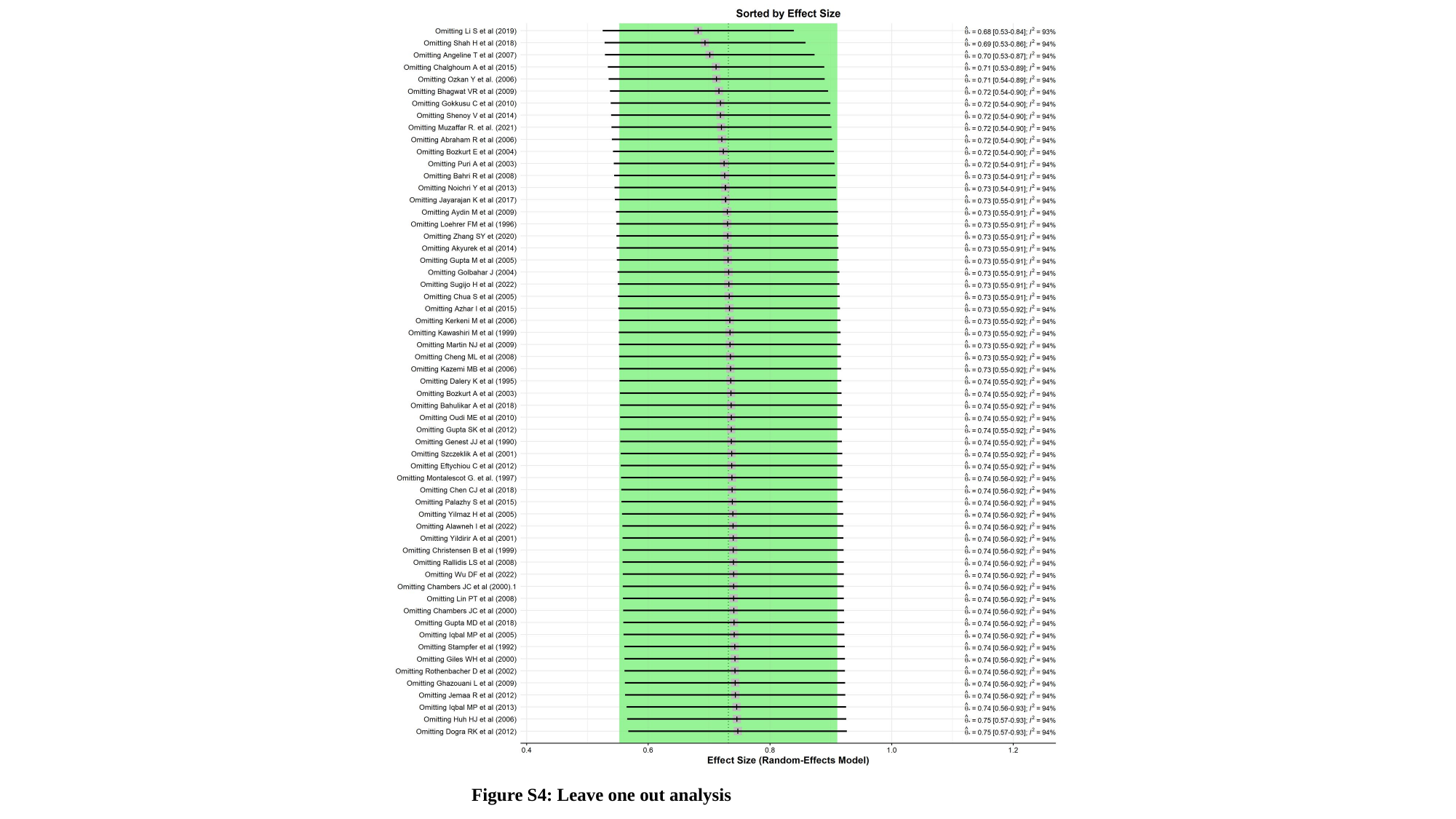

Figure S4: Leave one out analysis
